# Preventive Effect of Fisetin on Follicular Granulosa Cells Senescence via Attenuating Oxidative Stress and Upregulating the Wnt/β-Catenin Signaling Pathway

**DOI:** 10.3390/cells14211704

**Published:** 2025-10-30

**Authors:** Juan Dong, Zhaoyu Yang, Qiongyu Yuan, Weidong Zeng, Yuling Mi, Caiqiao Zhang

**Affiliations:** 1 College of Animal Sciences, Zhejiang University, No. 866 Yuhangtang Road, Hangzhou 310058, China; dongjuan502@zafu.edu.cn (J.D.); 12117050@zju.edu.cn (Z.Y.); qiongyu@zju.edu.cn (Q.Y.); zengwd@zju.edu.cn (W.Z.); 2 College of Animal Science and Technology & College of Veterinary Medicine, Zhejiang A&F University, No. 666, Wusu Road, Hangzhou 311300, China

**Keywords:** fisetin, chicken, follicular atresia, oxidative stress, β-catenin

## Abstract

**Highlights:**

**What are the main findings?**

- Fisetin alleviates oxidative damage and promotes ATP production in D-gal-induced senescent granulosa cells by activating the Nrf2/HO-1 signaling pathway.
- Fisetin mitigates cell cycle arrest and downregulates senescence marker expression in D-gal-treated granulosa cells via β-catenin nuclear translocation.

**What are the implications of the main findings?**

- The inhibition of Nrf2/HO-1 and β-catenin signaling abolishes the beneficial effects of fisetin on atresia of naturally aged follicles.
- Fisetin attenuates prehierarchical follicular atresia in aged laying hens by protecting granulosa cell function, supporting its potential as a nutritional intervention to delay ovarian aging.

**Abstract:**

Oxidative stress-mediated dysfunction of granulosa cells (GCs) is recognized as a pivotal driver of prehierarchical follicular atresia in poultry, contributing substantially to the reduced egg production in aged laying hens. Here, we investigated the protective effects of the natural flavonol, fisetin, on aged chicken follicular GCs. A D-galactose (D-gal)-induced aging model of GCs was established to evaluate the protective role of fisetin against cellular senescence. Small yellow follicles (SYFs) from 580-day-old hens were cultured with fisetin for 72 h to verify its ameliorative effect on naturally aged follicles. Fisetin reduced the typical characteristic of senescence in D-gal-induced GCs, as reflected by decreased senescence-associated β-galactosidase (SA-β-gal) activity and increased expression of proliferation-related proteins, including cyclin D1 (CCND1), cyclin-dependent kinase 2 (CDK2), cyclin-dependent kinase 1 (CDK1), and Cyclin B1. Furthermore, fisetin enhanced the activity of antioxidant enzymes by activating the Nrf2/HO-1 signaling pathways, while attenuating mitochondrial dysfunction and promoting ATP production in senescent GCs. Additionally, fisetin significantly promoted nuclear translocation of β-catenin, and suppressed the expression of senescence marker proteins p53 and p21, thereby alleviating cell cycle arrest in D-gal-induced senescent GCs. Simultaneous inhibition of Nrf2/HO-1 and β-catenin signaling also abolished the beneficial effects of fisetin on oxidative stress and cell proliferation in naturally senescent follicles. These findings indicate that fisetin prevents follicular atresia by suppressing GCs oxidative damage and improving cell cycle arrest via activating the Nrf2/HO-1 and Wnt/β-catenin signaling pathways.

## 1. Introduction

Ovarian follicles are the basic functional units of the chicken ovary and are essential for egg production. The rate of follicular atresia rate increases in the aging hens, primarily due to the diminished function of follicular GCs [1]. As the main functional cells within follicles, GCs regulate follicular activation, development, and maturation by mediating the transmission of energetic substances, hormones, and signaling molecules through gap junctions with oocytes. Oxidative stress, caused by an imbalance between the generation and elimination of reactive oxygen species (ROS), has been identified a key cause of GC damages and a major factor promoting pre-hierarchical follicular atresia in chickens [2]. Within follicles, GCs are particularly sensitive to elevated ROS levels. Accumulation of free radicals triggers mitochondrial damage, which acts as a key signal inducing cell cycle arrest in GCs, primarily due to impaired energy production from by dysfunctional mitochondria [3]. Concurrently, ROS initiate a strong DNA damage response that stabilizes p53, upregulates the cyclin-dependent kinase inhibitor p21, and ultimately induces cell cycle arrest. While cell cycle arrest facilitates cell damage repair, severe or prolonged arrest can trigger maladaptive repair processes or programmed cell death, including apoptosis [4]. Thus, suppressing oxidative stress in GCs represents a promising strategy to enhance cell survival and proliferation, thereby mitigating the decline in follicular quality associated with aging.

In recent years, numerous plant-derived natural compounds with antioxidant properties have emerged as potential agents for alleviating ovarian functional decline in aged laying hens. For instance, nobiletin [5], the polyphenol ellagic acid [6], and quercetin [7] have been demonstrated to mitigate oxidative stress in the ovarian tissue of aged laying hens, thereby reducing pre-hierarchical follicular atresia and maintaining high egg production performance. Fisetin (3,3′,4′,7-tetrahydroxyflavone), a bioactive flavonoid abundantly present in various fruits and vegetables such as persimmons, mangoes, grapes, apples, strawberries, peaches, cucumbers, onions, and tomatoes, has been widely investigated for its protective effects in various diseases owing to its multiple pharmacological activities, including antioxidant, anti-inflammatory, and anticarcinogenic properties, as well as its favorable safety profile [8]. Fisetin exerts its antioxidant effect by effectively scavenging ROS, maintaining the protective function of the nonenzymatic defense system glutathione (GSH), and ultimately reducing cell oxidative damage. For instance, in aged mice, intermittent oral fisetin supplementation improved vascular endothelial function by reducing mitochondrial ROS levels in the aorta, an effect mediated by decreased pro-oxidant signaling molecules and enhanced antioxidant defenses such as MnSOD [9]. Moreover, supplementation with fisetin protected porcine early embryos from oxidative stress during in vitro culture by lowering ROS levels and promoting GSH production [10]. Our previous study demonstrated that dietary fisetin supplementation restored ovarian antioxidant capacity and improved energy metabolism, thereby increasing egg production and reducing follicular atresia in aged laying hens [11]. However, the mechanisms by which fisetin alleviated follicular atresia of aged laying hens still remain to be further elucidated.

The Wingless/Int-1 (Wnt)/β-catenin signaling pathway, a highly conserved cascade composed of a range of proteins such as Wnt1, glycogen synthase kinase 3β (GSK3β), and β-catenin, plays a major role in regulating GC proliferation, differentiation, and apoptosis, which are directly associated with follicular development and atresia during ovary development [12]. β-catenin, encoded by catenin beta 1 (*CTNNB1*), functions as a key effector in the canonical Wnt signaling pathway. An in vitro study showed that activation of the Wnt/β-catenin pathway counteracted the reduced proliferation and viability induced by elevated fatty acids levels in human ovarian GCs, thus potentially improving the quality of growing follicles in women with obesity [13]. In mouse ovaries, β-catenin expressed in pre-GCs induces a morphological transition from squamous to cuboidal cells, an essential step supporting GC proliferation and differentiation during follicles activation [14]. Furthermore, β-catenin has been evidenced to promote CCND1 expression which in turn accelerates cell cycle progression in granulosa cells of hierarchical follicles in laying hens [15]. Collectively, these findings suggest that targeting the Wnt/β-catenin pathway may represent a promising therapeutic strategy to alleviate cell cycle arrest in GCs within hierarchical follicles and promote follicular development in aged laying hens.

The D-gal-induced cellular senescence models have been widely applied to investigate mechanisms of cellular senescence and anti-aging efficacy of various compounds [16,17]. Mechanistically, D-gal induces cellular damage through multiple pathways, including increased oxidative stress and disruption of autophagy flux and mitochondrial function [18]. These D-gal-induced cellular alterations closely resemble the main characteristics of natural cellular aging. In addition, our previous study demonstrated that D-gal exposure induces noticeable aging-related damage in cultured chicken ovaries tissues, such as increased follicular atresia, accumulation of ROS, and reduced ovarian cell proliferation [1]. Here, D-gal was used as an inducer of GCs senescence. This study aims to investigate the protective effects of fisetin against D-gal-induced senescence in follicular GCs of laying hens, as well as the underlying molecular mechanisms.

## 2. Materials and Methods

### 2.1. Animals and Samples Collection

Hy-line white chickens (*Gallus domesticus*) were purchased from a local commercial farm and raised with free access to feed and water. Ovaries were isolated from hens approximately 280 days old (high-laying period) and 580 (low-laying period) days old, respectively, and stored in the ice-cold sterile phosphate-buffered saline (PBS). The prehierarchical follicles (SYFs, 6–8 mm), ovarian cortex, and atretic small yellow follicles (ASYFs) were separated from the collected ovaries, and fixed in 4% paraformaldehyde (PFA) for morphological examination. Specifically, the ASYFs were collected from the 580-day-old hens. A portion of the SYFs from both age groups were used for granulosa layer isolation. Briefly, the SYFs were washed with cold-PBS, punctured with the tweezers, and the yolk was removed as clean as possible. The remaining granulosa layers were gently stripped from the theca layers, washed three times using PBS, and used for subsequent Western blot or quantitative real-time polymerase chain reaction (qRT-PCR) analysis. All procedures were approved by the Committee on the Ethics of Animals Experiments of Zhejiang University and conducted in accordance with guidelines the Guiding Principles for the Care and Use of Laboratory Animals of Zhejiang University (ZJU20220085).

### 2.2. Cell Culture and Treatments

The granulosa layers stripped from the SYFs of D280 hens were digested with collagenase 2 (Gibco, Grand Island, NY, USA), filtered through a 200-mesh steel sieve (75 μm) and centrifuged at 1500 rpm for 5 min. After washed with PBS twice, the cell mass was resuspended, counted, and cultured with DMEM/F12 medium (Hyclone, Tauranga, New Zealand) containing with 5% fetal calf serum (FCS, Hyclone, Logan, UT, USA) and 1% penicillin/streptomycin. The cells were incubated at 38 °C and in 5% CO_2_ to allow cell attachment overnight. After attachment, GCs were treated with different concentrations of fisetin (0, 2.5, 5, 10, 20, 40, 80 µM, MB5836, Meilunbio, Dalian, China) for 24 h to test the effect of fisetin on cell viability. To examine the anti-aging effect of fisetin on granulosa cells, the cells were pretreated with fisetin (0, 5, 10, 20, 40 µM) for 24 h, followed by incubation with the D-gal (200 mM, MB1853-2, Meilunbio, Dalian, China) for another 24 h. For inhibitor experiments, GCs were pretreated with each specific inhibitor for 2 h before fisetin exposure. ML385 (10 µM, HY-100523, MedChemExpress, Nanjing, China), the Nrf2 inhibitor and IWR-1 (10 µM, HY-12238, MedChemExpress), the β-catenin inhibitor, were used in these experiments.

### 2.3. Culture of Follicles and Treatment of Chemicals

SYFs from late-laying (aged 580 days) hens were transferred into DMEM/F12 complete medium (Hyclone) supplemented with 5% FCS (Hyclone) and 1 x ITS mixture (10 mg/mL insulin, 5 mg/mL transferrin, 30 nM selenite, and 2 mM glutamine). The follicles were cultured in 48-well plates (Corning Inc., Corning, NY, USA) at 38 °C and in a 5% CO_2_ atmosphere for 72 h. The treatment groups were designed as follows: fisetin (20 µM), fisetin + ML385 (10 µM), fisetin + IWR-1 (10 µM), ML385 (10 µM), and IWR-1 (10 µM). After 48 h of culture, the medium was supplemented with bromodeoxyuridine (BrdU, Sigma Aldrich, Saint Louis, MO, USA) at a final concentration of 10 µg/mL, followed by a further 24 h incubation.

### 2.4. Cell Viability Assay

Cell viability was assessed using a Cell Counting Kit-8 (CCK-8, FD3788, Fudebio, Hangzhou, China) according to the manufacturer’s instructions. Briefly, GCs were grown in 96-well plates and cultured for 24 h until reaching approximately 90% confluency, then treated with experimental conditions. For the assay, 10 µL of CCK-8 solution was added into each well containing 100 µL medium, and incubated for 2 h. Absorbance was measured at 450 nm using a microplate reader (Bio-Rad, Hercules, CA, USA).

### 2.5. SA-β-Gal Staining

Cellular senescence of D-gal-induced GCs was detected using a β-galactosidase staining kit (G1580, Solarbio, Beijing, China) according to the kit’s protocols. Briefly, after being incubated, the GCs were washed twice with PBS and fixed in β-galactosidase fixative solution for 15 min at room temperature. Following three washes with PBS, the cells were incubated in 1 mL dyeing liquid (10 µL β-galactosidase staining fluid A, 10 µL fluid B, 930 µL fluid C, and 50 µL X-Gal solution) overnight at 37 °C. The cells were observed and imaged using an Eclipse 80i microscope (Nikon, Tokyo, Japan). The cytoplasm of SA-β-gal-positive cells appeared as blue uniform particles. For each experimental condition, at least five randomly selected microscopic fields were analyzed, and the number of positive-stained cells per 200 cells was quantified using ImageJ v2.3.0 software (NIH, Bethesda, MD, USA) to distinguish blue cells (positive) from unstained cells (negative) by setting a color threshold. All experiments were performed in triplicate.

### 2.6. Cell-Cycle Distribution

The effects of different treatments on cell cycle progression were analyzed by flow cytometry. Briefly, GCs were plated at 1 × 10^6^ cells per 60 mm dish. After indicated treatments, the cells were trypsinized, washed with PBS, and fixed with 75% ethanol on ice followed by resuspension in 500 µL of propidium iodide solution (480 µL staining buffer, 10 µL PI, and 10 µL RNase A) and incubated at 37 °C for 30 min. Cell cycle distribution were assayed using a FACSCalibur flow cytometer (BD Biosciences, Franklin Lakes, NJ, USA) with an extraction wavelength of 488 nm. Cell cycle distribution was analyzed using FlowJo v10.8.1 software (FlowJo, LLC, Ashland, OR, USA).

### 2.7. The 5-Ethynyl-2′-deoxyuridine (EdU) Assay

Cell proliferation was assessed using an EdU assay kit (C0071S, Beyotime, Hangzhou, China) according to the manufacturer’s protocol. GCs were incubated with 10 µM EdU for 2 h at 38 °C. The treated GCs subsequently underwent fixation with 4% PFA for 15 min at room temperature and permeabilized with into 0.5% Triton X-100 for 15 min. After three washes with PBS, the GCs were incubated with 0.5 mL click reaction solution (430 µL click reaction buffer, 20 µL CuSO_4_, 1 µL Azide 594, and 50 µL click additive solution) for 30 min in the dark. Nucleic acids were marked using DAPI (C1006, Beyotime, Hangzhou, China). Images were captured by a fluorescence microscope (Olympus IX70, Tokyo, Japan). All experiments were performed in triplicate.

### 2.8. Determination of Mitochondrial Membrane Potential (MMP)

Mitochondrial membrane potential (MMP), an indicator of mitochondrial inner and outer membrane integrity, was estimated using an enhanced mitochondrial membrane potential assay kit with the fluorescent probe JC-1 (C2003S, Beyotime) following the instruction. Cells were plated in 6-well culture plates at a density of 1 × 10^6^ cells per well and allowed to adhere for 24 h. After the indicated treatment, GCs were subjected to two PBS rinses and then incubated with 1 mL of JC-1 working solution per well at 38 °C for 20 min. Subsequently, cells were rinsed twice with premade JC-1 staining buffer and analyzed for red and green fluorescence signals using either laser confocal microscopy (Olympus IX81-FV1000, Tokyo, Japan) or flow cytometry. Quantification of red and green fluorescence was performed using ImageJ (v2.3.0, NIH, Bethesda, MD, USA), and the ratio was calculated from the mean optical density of each. FlowJo v10.8.1 software was performed to analyze the flow cytometry.

### 2.9. Intracellular ROS Assessment

Intracellular ROS was measured using a ROS Assay Kit (S0033S, Beyotime) according to the manufacturer’s instructions. Briefly, after exposure to fisetin and D-gal, GCs were collected and incubated with 10 µM fluorescent probe 2′,7′-dichlorofluorescein diacetate (DCFH-DA) for 20 min at 38 °C. Following two washes with pre-cooled PBS twice, fluorescence was measured by flow cytometry with excitation at 488 nm and emission fluorescence at 525 nm.

### 2.10. Measurement of Intracellular Catalase (CAT), Total Superoxide Dismutase (T-SOD), Malonaldehyde (MDA), Glutathione (GSH), and Adenosine Triphosphate (ATP)

After treatments, cells or SYFs were harvested to assess the intracellular levels of CAT, T-SOD, MDA, GSH, and ATP. For the measurement of oxidative parameters in the cultured ovarian follicles, tissues were homogenized in PBS and then centrifuged at 2500 rpm for 10 min at 4 °C to obtain a 10% tissue homogenate. The supernatants were used for the determination of total protein concentration and subsequent measurements of oxidative parameters. Total protein concentration, CAT, T-SOD activities, and the levels of MDA, GSH, and ATP were measured according to the manufacturer’s instruction using kits (Nanjing Jiancheng Bioengineering Institute, Nanjing, China).

### 2.11. Histology and Immunohistochemistry (IHC)

The SYFs, ASYFs, ovarian cortex, and the cultured follicles were washed with PBS three times and fixed in 4% paraformaldehyde for 24 h at 4 °C. Samples were then dehydrated, embedded in paraffin, and sectioned at a thickness of 5 µm. Hematoxylin and eosin (H&E) staining was performed following standard procedures. For IHC staining, paraffin sections were deparaffinized in xylene, rehydrated through a graded alcohol series, and treated with 3% hydrogen peroxide to block endogenous peroxidase activity. Antigen retrieval was conducted according to standard protocols. The sections were washed three times using PBS and then blocked by 10% normal goat serum for 20 min. Subsequently, the slides were incubated overnight at 4 °C with primary antibody of rabbit anti-p53 (1:50, ET7107-33, Huabio, Hangzhou, China). After washing with PBS three times, sections were incubated with goat anti-rabbit IgG-HRP (1:500, HA1001, Huabio) at 37 °C for 1 h. Immunoreactivity was visualized using DAB substrate, followed by hematoxylin counterstaining for nuclear visualization. Images were captured using an Eclipse 80i microscope (Nikon).

### 2.12. Immunofluorescence (IF)

The chicken follicular slides were deparaffinized, hydrated, and subjected to antigen retrieval following established laboratory procedures. GCs were seeded onto sterilized glass coverslips placed in 24-well plates and cultured according to a standard protocol. After the indicated treatments, the cell-covered coverslips were washed with PBS and fixed in 4% paraformaldehyde for 10 min. Samples were then washed three times with PBS, permeabilized with 0.5% Triton X-100 for 10 min, and then blocked with 10% normal goat serum for 20 min at room temperature. The follicular sections and cell coverslips were incubated overnight at 4 °C with anti-β-catenin (1:50, Huabio), anti-BrdU antibody (1:200, G3G4; DSHB, Iowa City, IA, USA), or anti-Lamin B1 (1:100, ET1606-27, Huabio). After washing three times with PBS, samples were incubated with goat anti-rabbit Alexa Fluor 594 (1:500, A11037, Abclonal, Wuhan, China) for 1 h at 37 °C in the dark. Nuclei was stained with DAPI (Beyotime) for 5 min at room temperature. Images were captured using laser confocal microscopy (Olympus IX81-FV1000).

### 2.13. Western Blot (WB) Analysis

Western blotting was performed using the conventional protocol. Briefly, total protein from tissues and cells was extracted on ice using RIPA buffer (P0013B, Beyotime) supplemented with phosphatase inhibitor cocktail. Protein concentration was determined with a BCA protein assay kit (P0009, Beyotime). Cytoplasmic and nuclear extracts were prepared using the Nuclear Protein Extraction Kit (R0050, Solarbio, Beijing, China) following the manufacturer’s instructions. Protein samples were diluted to 2 µg/µL using loading buffer and denatured at 100 °C for 10 min. Equal amounts (10 µL) of protein were loaded onto a 10% SDS-polyacrylamide gel (8.6 cm × 6.8 cm × 1.0 mm) prepared using the SDS-PAGE Gel Preparation Kit (FD2190, Fudebio) and separated at a constant voltage of 200 V for 1 h. The separated proteins were electrotransferred onto nitrocellulose membranes (Millipore, Darmstadt, Germany) at a constant current of 200 mA for 45 min at room temperature. Membranes were blocked with 5% non-fat milk for 1 h at room temperature, then incubated overnight at 4 °C with the following primary antibodies: CCND1 (1:500, ER0722, Huabio), CDK2 (1:500, R1309-3, Huabio), CDK1 (1:500, ER31213, Huabio), Cyclin B1 (1:500, R1308-13, Huabio), Caspase-3 (1:500, ER1802-42, Huabio), cleaved Caspase-3 (Asp175) (1:500, 9664, Cell Signaling Technology, Beverly, MA, USA), TOMM20 (1:1000, ET1609-25, Huabio), HO-1 (1:500, ER1802-73, Huabio), NQO1 (1:500, ER1802-85, Huabio), Nrf2 (1:500, ER1706-41, Huabio), phospho-Nrf2 (1:1000, ET1608-28, Huabio), Lamin B1 (1: 1000, Huabio), p53 (1:500, Huabio), CDKN1A/p21 (1:500, HA500156, Huabio), GSK3 beta (1:500, ET1607-7, Huabio), phospho-GSK3 beta (1:500, ET1607-60, Huabio), β-catenin (1:500, Huabio), Histone H3 (1:500, R1105-1, Huabio), and β-actin (1:5000, EM2001-07, Huabio). Following three washes with TBST, the membranes were probed with an HRP-conjugated secondary antibody for 1 h at room temperature. Immunoreactive bands were visualized using clarity ECL Western blot substrate kits (Bio-Rad, #1705061, Hercules, CA, USA) and imaged with a ChemiScope 3400 Mini machine (Clinx, Shanghai, China). Band intensities were quantified using ImageJ v2.3.0 software (NIH), and target protein expression levels were normalized to β-actin as control.

### 2.14. RNA Extraction and qRT-PCR

Total RNA was extracted from tissues using Trizol reagent. Reverse transcription was conducted using the HiScript II 1st Strand cDNA Synthesis Kit (R211-01, Vazyme, Nanjing, China) with 1 µg of total RNA in accordance with the manufacturer’s instructions. The qRT-PCR was performed to assess the expression of gene which involved in glycolysis by using a HiScript II One Step qRT-PCR SYBR Green Kit (Q221-01, Vazyme, Nanjing, China). The primers used in the PCR are listed in Table S1. The relative mRNA expression levels were calculated using the 2^−∆∆Ct^ method, with β-actin serving as the internal reference gene.

### 2.15. Transmission Electron Microscopy (TEM)

The cultured SYFs were collected and fixed in 2.5% glutaraldehyde overnight at 4 °C, followed by post-fixed in buffered 1% osmium tetroxide for 1.5 h. Sample were then dehydrated through a grade series of ethyl alcohol or acetone, and eventually wrapped in epoxypropane resin according to the standard TEM procedures. Ultrathin sections (70–90 nm) were obtained using an ultramicrotome (Leica EM UC7, Wetzlar, Germany), stained with 8% aqueous uranyl acetate and Reynold’s lead citrate, and observed under a Tecnai G2 Spirit (FEI Company, Hillsboro, OR, USA) with an acceleration voltage of 120 kV at various magnifications.

### 2.16. Statistical Analysis

All data are presented as the mean ± SEM. *T*-test was used to assess the difference between two samples. Statistical differences between groups were analyzed and determined by one-way analysis of variance (ANOVA) followed by Tukey’s test or Dunnett’s test. The results were regarded as significant when *p* < 0.05.

## 3. Results

### 3.1. Fisetin Postpones GCs Senescence Induced by D-Gal

**Figure 1:**
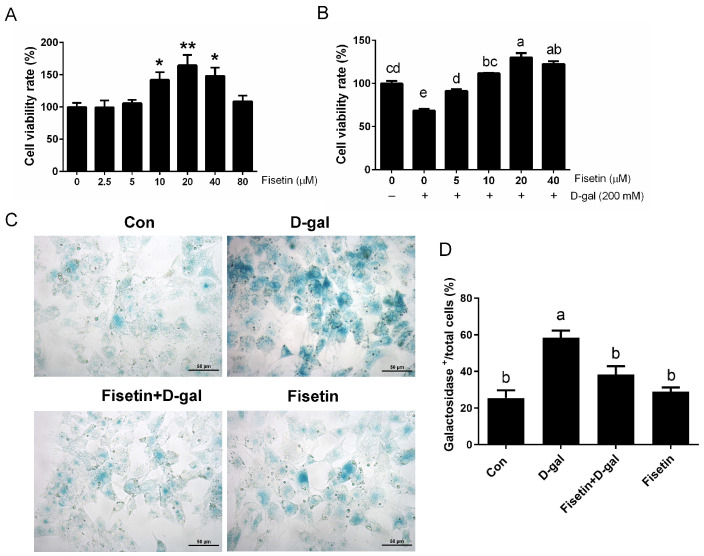
Fisetin inhibits D-gal-induced senescence in SYF-GCs. (**A**) Effect of different concentrations of fisetin on SYF-GC viability as determined by CCK-8 (*n* = 6). (**B**) Effects of fisetin and 200 mM D-gal treatment on SYF-GC proliferation as assessed by the CCK-8 assay. (**C**,**D**) Quantification and representative images of SA-β-gal-positive cells among total GCs following the indicated treatments. Scale bar: 50 μm. * *p* < 0.05, ** *p* < 0.01 indicate statistically significant difference relative to control, following fisetin treatment of different concentrations for 24 h. Different lowercase letters indicate significant differences (*p* < 0.05).

As shown in Figure 1A, compared with the control group, GCs exposed to 10 µM, 20 µM and 40 µM fisetin for 24 h exhibited a significant increase in cell viability, with 20 µM concentration displaying the highest effect. As expected, treatment with 20 µM fisetin significantly restored the viability of GCs following D-gal exposure (Figure 1B). β-galactosidase staining, a widely used approach for detecting cellular senescence, further confirmed these findings. is the most commonly used approach for detecting senescent cells. As presented in Figure 1C,D, pretreatment with 20 µM fisetin markedly reduced the proportion of β-galactosidase-positive cells. These results demonstrated that fisetin reversed the D-gal-induced senescence in SYF-GCs.

### 3.2. Fisetin Alleviates Cell-Cycle Arrest Caused by D-Gal

**Figure 2:**
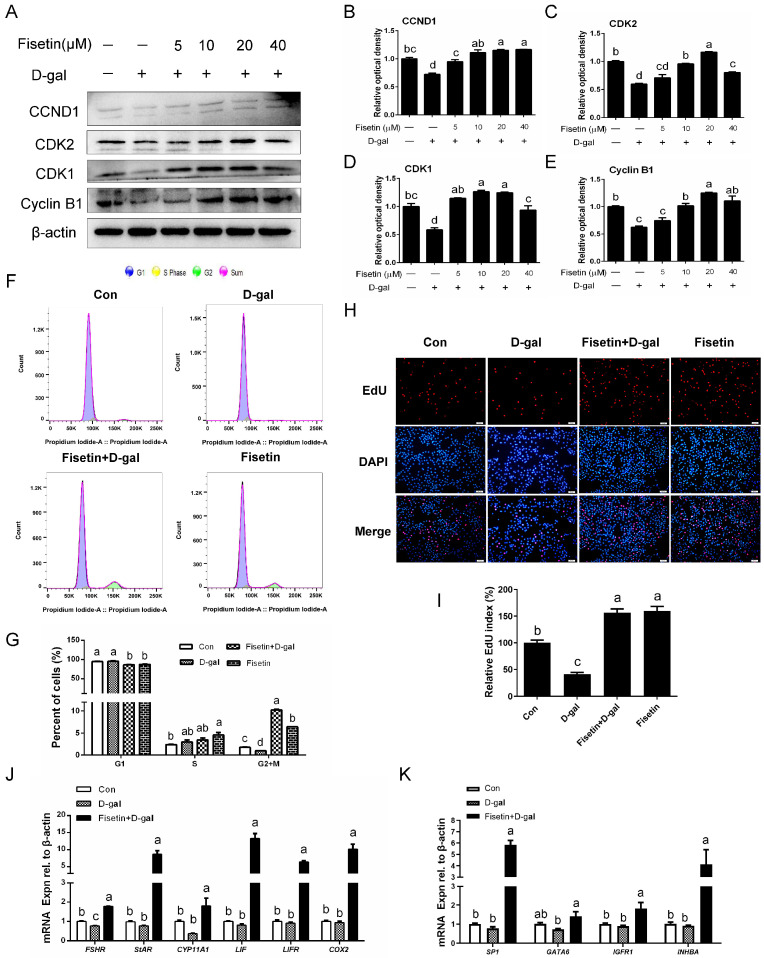
Effect of fisetin on D-gal-induced impairment of proliferation and cell cycle arrest in SYF-GCs. SYF-GCs were pretreated with fisetin (5 µM, 10 µM, 20 µM, and 40 µM) for 24 h, and the co-cultured with D-gal (200 mM) for another 24 h. (**A**–**E**) The protein levels of CCND1, CDK2, CDK1, and Cyclin B1 in cultured GCs were analyzed by Western blotting, with β-actin used as a loading control. (**F**,**G**) Flow cytometric analysis of cell-cycle distribution in GCs following treatments of control, D-gal (200 mM) for senescence, D-gal with fisetin (20 µM) pretreatment, and fisetin alone. (**H**,**I**) EdU assay in GCs under the indicated treatment. Scale bar: 50 μm. (**J**,**K**) qRT-PCR analysis of *FSHR*, *StAR*, *CYP11A1*, *LIF*, *LIFR*, *COX2*, *SP1*, *GATA6*, *IGFR1*, and *INHBA* mRNA levels in the cultured GCs. Different lowercase letters indicate significant differences (*p* < 0.05).

Both replicative and premature senescent cells exhibit significant proliferation inhibition. As shown in Figure 2A–E, D-gal markedly decreased the expression of the proliferation-related proteins CCND1, CDK2, CDK1, and Cyclin B1, whereas fisetin pretreatment effectively reversed these alterations. Specifically, CCND1 expression was elevated in the groups co-treated with D-gal and fisetin at doses of 5, 10, 20, and 40 µM compared with the D-gal group, with the 5 and 10 µM combination groups showing no significant difference from the control. The D-gal-induced downregulation of CDK2 was significantly reversed by fisetin at 10, 20, 40 µM, although expression in the 40 µM fisetin and D-gal combination group did not reach control levels. Similarly, CDK1 expression was elevated in the 5, 10, 20, and 40 µM fisetin-pretreated groups relative to the D-gal alone, except in the 5 and 40 µM groups, were comparable to the control. Consistently, Cyclin B1 levels exhibited a dose-dependent upregulation in D-gal-induced SYF-GCs with increasing concentrations of fisetin from 10 to 40 µM. Our previous findings further suggested cellular senescence by demonstrating a marked G1 phase arrest in D-gal-treated SYF-GCs, aligning with the key feature of cell cycle arrest [1]. However, fisetin pretreatment effectively alleviated this cell-cycle arrest triggered by D-gal -induced damage (Figure 2F,G). Furthermore, fisetin mitigated the reduction in EdU-labeling cells caused by D-gal treatment, thereby restoring SYF-GCs proliferation capacity (Figure 2H,I). The mRNA levels of follicle-stimulating hormone (*FSHR*), steroidogenic acute regulatory (*StAR*), cytochrome p450 family 11 subfamily A member1 (*CYP11A1*), leukemia inhibitory factor (*LIF*), leukemia inhibitory factor receptor (*LIFR*), cyclooxygenase 2 (*COX2*), specificity protein 1 (*SP1*), GATA binding 6 (*GATA6*), insulin-like growth factor 1 receptor (*IGFR1*), and inhibin activin subunit beta A (*INHBA*) in D-gal-induced senescent GCs were significantly upregulated after the addition of fisetin (Figure 2J,K).

### 3.3. Fisetin Protects GCs from D-Gal-Induced Cytotoxicity by Inhibiting Mitochondrial Injury

**Figure 3:**
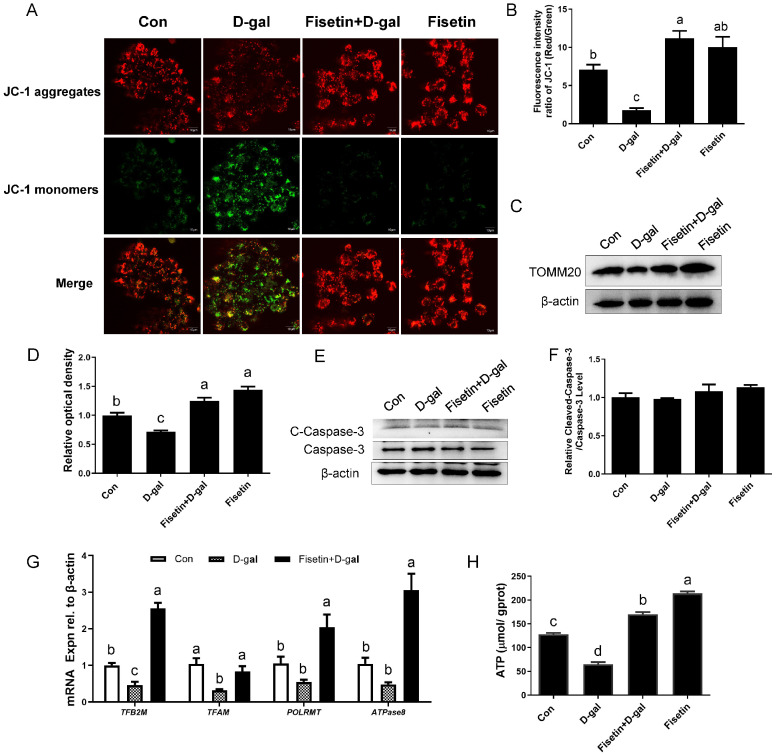
Fisetin attenuates the mitochondrial damage in D-gal-induced senescent GCs. (**A**,**B**) SYF-GCs were treated with fisetin (20 µM) for 24 h, followed by co-culture with D-gal (200 mM) for another 24 h. Mitochondrial membrane potential was assessed by fluorescence microscopy following staining with the JC-1 probe. Scale bar: 10 μm. (**C**,**D**) WB assay of TOMM20. (**E**,**F**) The protein level of cleaved-Caspase-3 and Caspase-3 was determined by Western blotting, and β-actin served as the control for loading. (**G**) Transcription levels of the *TFB2M*, *TFAM*, *POLRMT*, and *ATPase8* genes in control, D-gal-treated GCs, and fisetin + D-gal GCs. (**H**) Detection of ATP levels in GCs from the different treatment groups. Different lowercase letters indicate significant differences (*p* < 0.05).

The results of JC-1 staining revealed that compared with the control, D-gal-treated GCs showed reduced red fluorescence, enhanced green fluorescence and a decreased MMP, indicating mitochondrial dysfunction. The effect was effectively reversed in GCs pretreated with fisetin (Figure 3A,B). Similarly, Western blot analysis showed that TOMM20, a mitochondrial mass marker, was significantly upregulated in D-gal-induced senescent GCs following fisetin treatment. GCs treated with fisetin alone also exhibited higher TOMM20 expression compared to the control group (Figure 3C,D). Notably, GCs co-exposed to D-gal and fisetin exhibited a low cleaved-caspase-3 (C-Caspase-3)/Caspase-3 ratio, indicating that the resulting mitochondrial damage occurred independently of apoptosis in GCs (Figure 3E,F). Pretreatment of fisetin further enhanced the mRNA levels of mitochondrial biogenesis-related genes, including mitochondrial transcription factor B2 (*TFB2M*), mitochondrial transcription factor A (*TFAM*), RNA polymerase, mitochondrial transcriptional (*POLRMT*), and ATP synthase membrane subunit 8 (*ATPase8*) in D-gal-induced senescent GCs (Figure 3G). In addition, the decreased ATP content in D-gal-induced senescent GCs was reversed by fisetin pretreatment, while fisetin administration alone significantly elevated ATP levels relative to the control (Figure 3H).

### 3.4. Fisetin Reduces D-Gal-Induced Oxidative Stress in GCs via Activation of Nrf2/HO-1 Pathway

**Figure 4:**
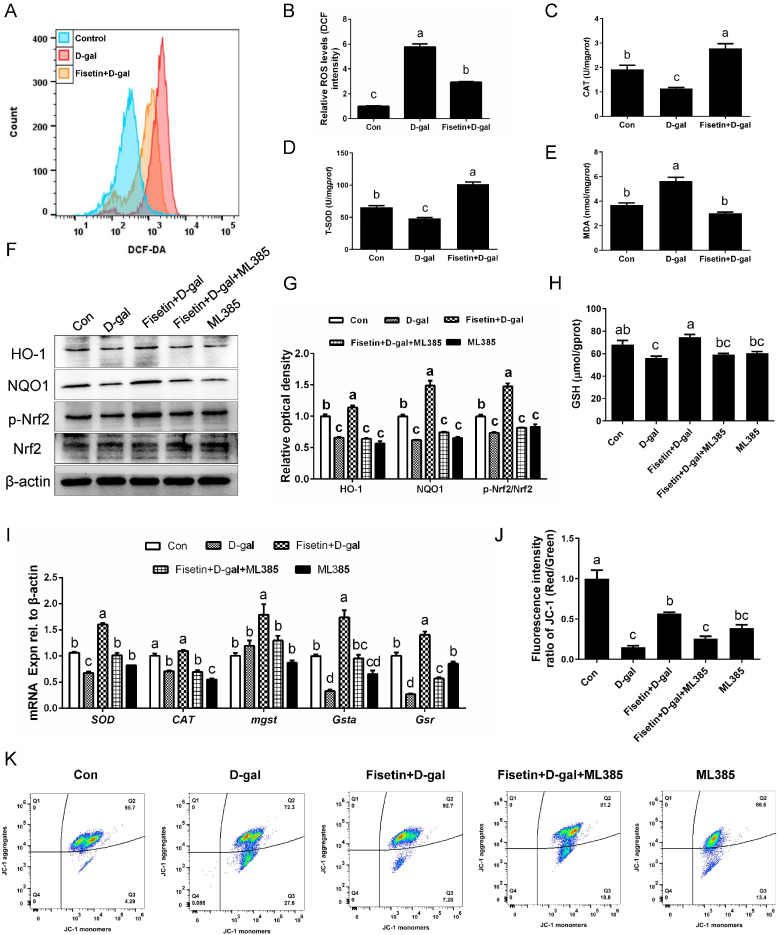
The protective effect of fisetin against D-gal-induced oxidative stress in SYF-GCs was mediated by the Nrf2/HO-1 signaling pathway. (**A**,**B**) GCs were pretreated with 20 µM fisetin for 24 h and then cultured with 200 mM D-gal for another 24 h. The intracellular ROS production was detected by flow cytometry. (**C**–**E**) Antioxidative levels (CAT and T-SOD) and oxidative product (MDA) in the GCs under the indicated treatments. (**F**,**G**) WB assay of HO-1, NQO1, p-Nrf2, and Nrf2 in control, D-gal, Fisetin + D-gal, ML385 alone and ML385 + fisetin +D-gal groups. β-actin served as an invariant control for equal loading. (**H**) Detection of GSH level. (**I**) The mRNA levels of the *SOD*, *CAT*, *Mgst*, *Gsta*, *Gsr* genes in GCs following treatments described in F. (**J**,**K**) Detection of MMP in flow cytometry. Different lowercase indicated significant differences (*p* < 0.05).

Flow cytometry analysis following DCF-DA staining showed that ROS levels were markedly enhanced in D-gal-induced senescent GCs, whereas fisetin pretreatment significantly attenuated this increase (Figure 4A,B). Similarly, the D-gal-induced reduction in antioxidant enzyme activities, including T-SOD and CAT, was effectively restored by fisetin pretreatment (Figure 4C,D). Consistent with these findings, MDA levels in the D-gal group were significantly higher than the other two groups (Figure 4E). WB assay showed that the protein levels of p-Nrf2/Nrf2, HO-1, and NQO1 were significantly decreased in D-gal-induced senescent GCs relative to control, and fisetin pretreatment prevented this decline effectively. To further elucidate the role of Nrf2 in mediating fisetin’s protective effects against D-gal-induced oxidative stress, Nrf2/HO-1 signaling was inhibited using the specific Nrf2 inhibitor, ML385. Pretreatment with ML385 blocked the fisetin-induced activation of Nrf2/HO-1 signaling and attenuated GSH activity in D-gal-induced senescent GCs (Figure 4F–H). At the transcriptional level, qRT-PCR analysis revealed that inhibition of Nrf2/HO-1 signaling reversed the fisetin-induced upregulation of antioxidant genes, including *SOD*, *CAT*, glutathione S-transferase alpha (*Gsta*), glutathione reductase (*Gsr*), and microsomal glutathione S-transferase (*Mgst*), in D-gal-induced senescent GCs (Figure 4I). In addition, the improvements in mitochondrial dysfunction observed in fisetin-treated senescent GCs were abolished by Nrf2/HO-1 inhibition (Figure 4J,K).

### 3.5. The Changes in Β-Catenin and Cell Cycle-Related Proteins in Ovaries, SYFs and ASYFs of D280 and D580 Hens

**Figure 5:**
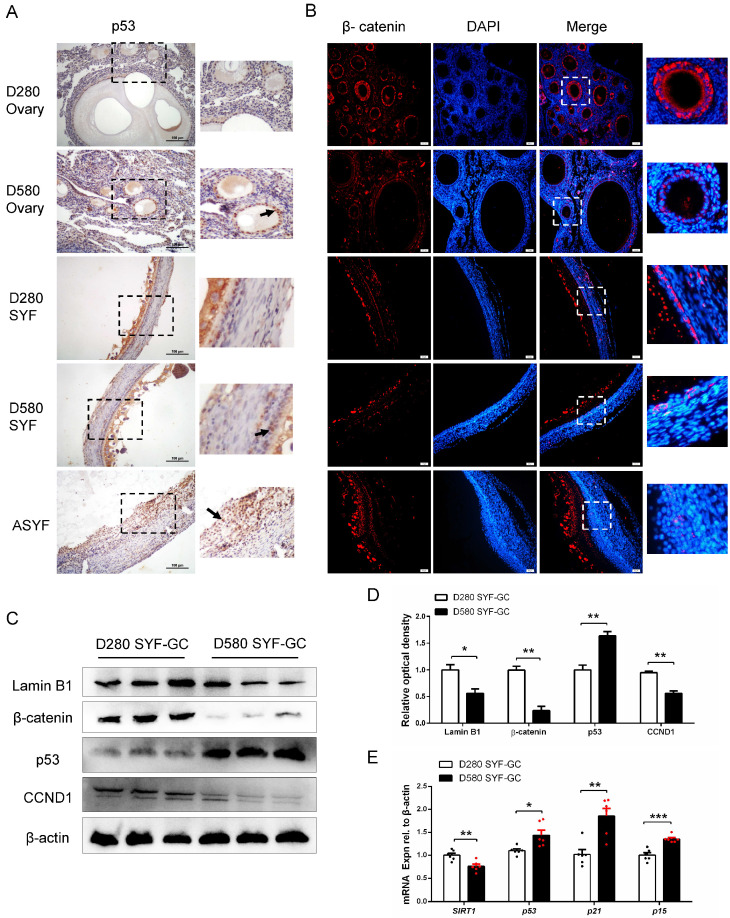
Expression of β-catenin and cell cycle-related proteins in ovarian and follicular tissues of D280 and D580 laying hens. (**A**) Expression of p53 in ovarian tissues and SYFs from D280 and D580 hens, and ASYFs visualized by IHC. Black arrows represent positive-colored GCs. Scale bar: 100 µm. (**B**) IF staining for β-catenin in the ovaries, SYFs, and ASYFs from D280 and D580 hens. Scale bar: 50 µm. (**C**,**D**) WB analysis of Lamin B1, β-catenin, p53, and CCND1 expression in granulosa cells of SFYs from D280 and D580 hens. (**E**) The mRNA levels of *SIRT1*, *p53*, *p21*, and *p15* were determined by qRT-PCR in GCs isolated from SYFs of D280 and D580 hens. * *p* < 0.05, ** *p* < 0.01, *** *p* < 0.001 represent the differences between SYF-GCs from 280-day-old hens and those from 580-day-old hens.

As shown in Figure 5A, p53 expression in the granulosa layers of ovarian follicles from D580 hens markedly higher than in D280 hens, except in the vitelline membrane, and was strongly expressed in the ASYFs. IF staining showed β-catenin was predominantly localized in the cytoplasm of GCs in SYFs and ovarian follicles from D280 hens. In contrast, β-catenin expression was markedly reduced in ovarian follicles and SYFs from D580 hens and was almost undetectable in ASYFs (Figure 5B). WB analysis indicated that Lamin B1, β-catenin, and the proliferation-related protein CCND1, were all significantly down-regulated in SYF-GCs at D580 hens compared to D280 hens, whereas p53 expression was markedly increased with aging. (Figure 5C,D). In addition, the senescence-related genes *p53*, *p21*, and *p15* mRNA levels showed a significant increase in SYF-GCs from D580 hens, while the gene of SIRT1 showed a down-regulation expression in SYF-GCs from D580 hens compared to that from D280 hens (Figure 5E).

### 3.6. Fisetin Rescues D-Gal-Induced Cell Cycle Arrest via β-Catenin Upregulation in GCs

**Figure 6:**
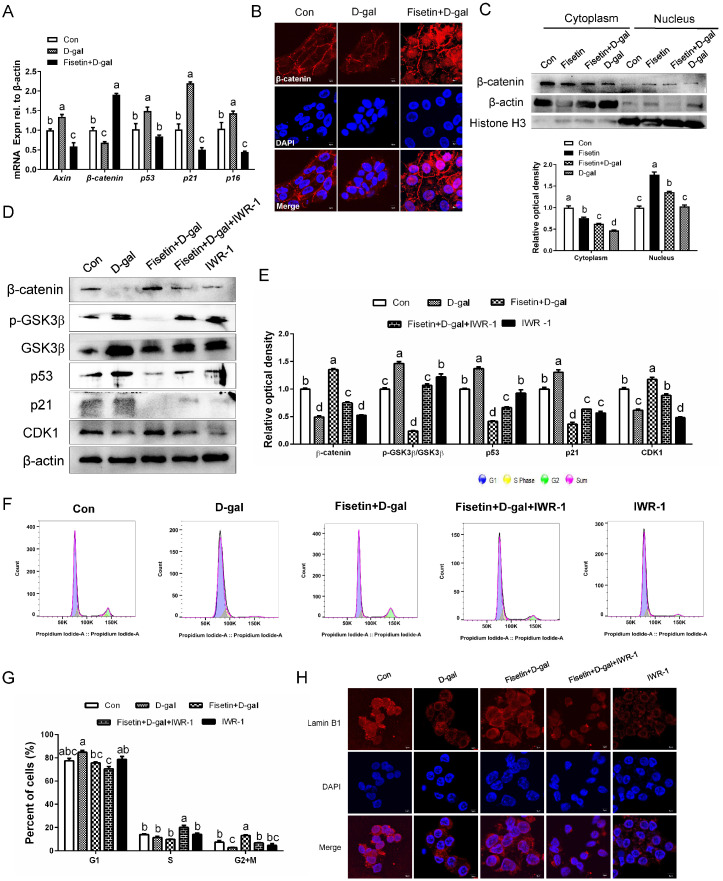
Fisetin alleviated D-gal-induced cell senescence and cell cycle arrest by upregulating β-catenin. (**A**) The mRNA levels of *Axin*, *β-catenin*, *p53*, *p21* and *p16* genes in the control, D-gal, Fisetin and D-gal combined treatment groups. (**B**) IF analysis of β-catenin in control GCs, senescent GCs (D-gal), and senescent GCs upon fisetin treatment (D-gal + Fisetin). Scale bar: 5 µm. (**C**) GCs were pretreated with 20 µM fisetin for 24 h and then cultured with 200 mM D-gal for another 24 h, the nuclear and cytoplasmic fractions were from GCs and analyzed by Western blotting using the indicated antibodies. (**D**,**E**) WB analysis of β-catenin, GSK3β, p-GSK3β, p53, p21 and CDK1 proteins in normal, D-gal, D-gal + Fisetin, D-gal + Fisetin + IWR-1 (β-catenin inhibitor), IWR-1 groups. (**F**,**G**) Cell cycle progression of treated GCs was analyzed by flow cytometry. (**H**) Representative images of Lamin B1 staining in senescent GCs under the indicated treatment conditions. Scale bar: 5 µm. Different lowercase letters indicate significant differences (*p* < 0.05).

Compared to the control, D-gal treatment significantly decreased β-catenin while increasing *Axin*. In contrast, fisetin partly restored this phenomenon (Figure 6A). Additionally, qRT-PCR analysis revealed that fisetin reduced the D-gal-induced upregulation of key senescence-related genes, namely *p53*, *p21*, and *p16*, in SYF-GCs. Fisetin treatment robustly promoted β-catenin accumulation in the nucleus, as evidenced by IF staining and confirmed through separate Western blot analysis of nuclear and cytoplasmic protein extracts (Figure 6B,C). To investigate the involvement of β-catenin in fisetin-mediated effects on GC proliferation and senescence, we employed the specific inhibitor IWR-1. Inhibition of β-catenin completely abolished the ability of fisetin to downregulate p53 and p21 expression, thereby also preventing the fisetin-induced increase in CDK1 activity in senescent GCs (Figure 6D,E). Correspondingly, IWR-1 treatment prevented fisetin from alleviating D-gal-induced cell cycle arrest (Figure 6F,G). Furthermore, the upregulation of Lamin B1 by fisetin was suppressed upon co-treatment with the β-catenin inhibitor (Figure 6H).

### 3.7. Blockage of Nrf2 and β-Catenin Expression Weakens the Role of Fisetin on Delaying Follicular Atresia in Low-Yield Laying Chickens

**Figure 7:**
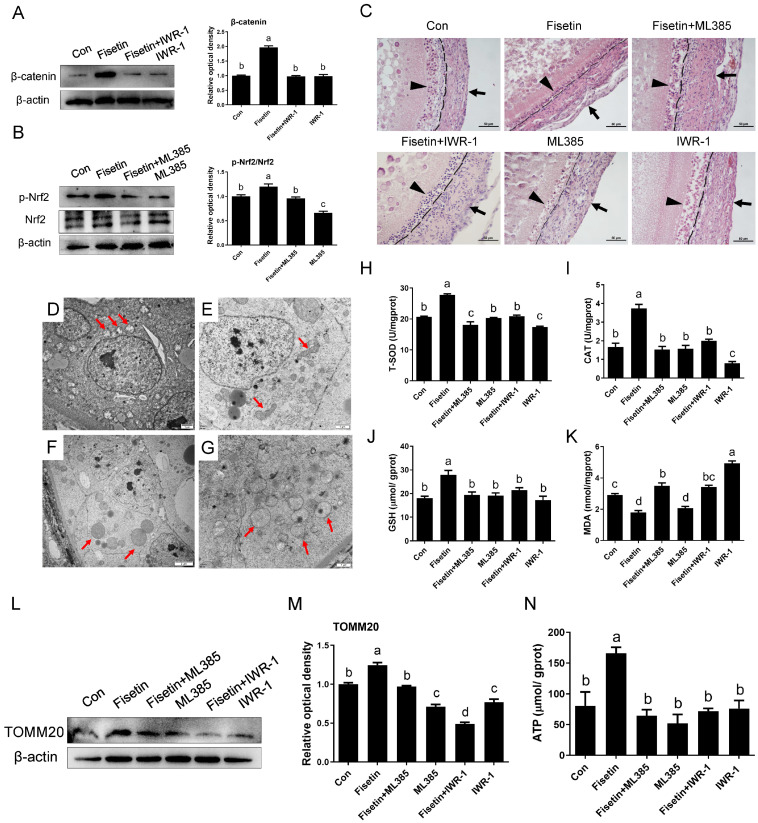
Fisetin attenuated the oxidative stress of SYFs in 580-day-old hens via regulating Nrf2 and β-catenin signaling pathways. (**A**,**B**) WB analysis of p-Nrf2, Nrf2, and β-catenin in control, Fisetin, Fisetin + ML385, Fisetin + IWR-1, ML385, IWR-1-treated groups. (**C**) H&E staining of SYFs following the indicated treatments. Scale bar: 50 µm. The granulosa layer (arrowheads) and theca layer (arrows) from follicles and atretic follicles were separated by a dashed line. (**D**–**G**) TEM observation of SYFs from control, Fisetin, Fisetin + ML385, and Fisetin + IWR-1-treated groups. Scale bar: 1 µm. Red arrows: mitochondria. (**H**–**K**) Determination of CAT, T-SOD, GSH, and MDA in SYFs of each group. (**L**,**M**) WB analysis of TOMM20 in SYFs of each group. (**N**) ATP detection. Different lowercase letters indicate significant differences (*p* < 0.05).

**Figure 8:**
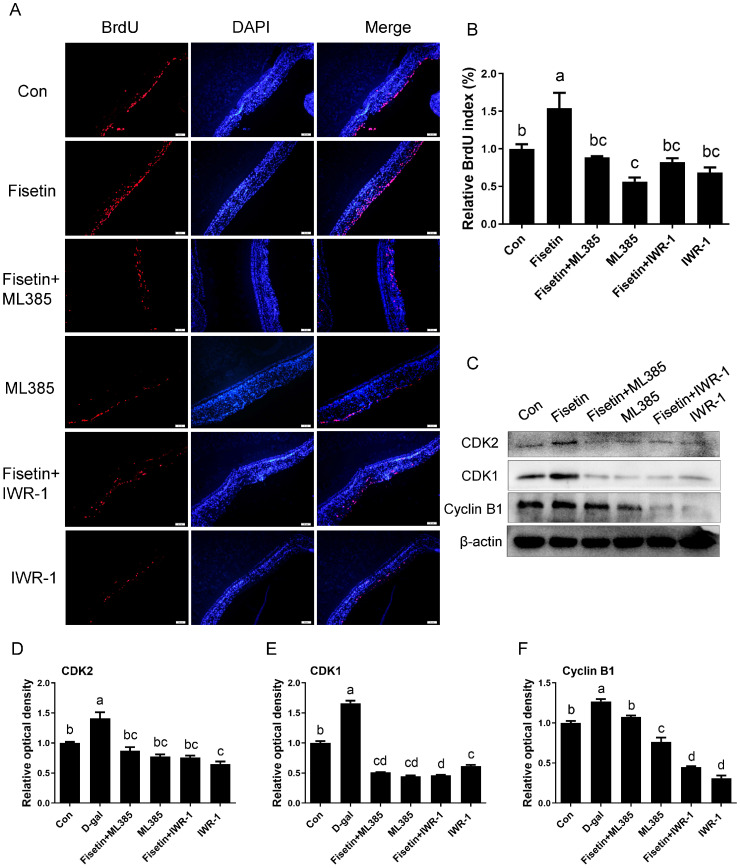
Protective effects of fisetin against aging in chicken SYFs were inhibited by the concurrent blockade of the Nrf2 and β-catenin signaling pathways. (**A**,**B**) BrdU staining of SYFs from D580 hens in control, fisetin group, fisetin and ML385 combined treatment group, fisetin and IWR-1 combined group, ML385 alone, and IWR-1 alone group. Scale bar: 100 µm. (**C**–**F**) WB analysis of CDK2, CDK1, and Cyclin B1 in SYFs from the indicated treatment groups. Different lowercase letters indicate significant differences (*p* < 0.05).

In naturally aged SYFs from D580 hens, fisetin treatment activated Nrf2 signaling and increased β-catenin expression, whereas the addition of the inhibitors ML385 (Nrf2 inhibitor) and IWR-1 (β-catenin inhibitor) reversed these effects (Figure 7A,B). Morphology observation indicated a notable promotion of follicular development and maintenance of structural integrity in SYFs by fisetin, as evidenced by tightly arranged granulosa and theca layers relative to the control group. Conversely, inhibition of Nrf2 and β-catenin expression concurrently disrupted GC integrity and altered follicle morphology with loosely and irregularly arranged granulosa layers (Figure 7C). TEM analysis confirmed that the inhibitor abolished the protective effect of fisetin on mitochondrial ultrastructure in GCs from D580-SYFs (Figure 7D–G). Biochemical analysis showed that fisetin increased the activities of T-SOD and CAT, as well as GSH content, and reduced MDA levels in D580 SYFs; these effects were abrogated by co-treatment with ML385 or IWR-1 (Figure 7H–K). Western blotting further confirmed the upregulation of TOMM20 by fisetin, which was effectively suppressed by the inhibitor (Figure 7L,M). Fisetin treatment increased ATP content in D580-SYFs, which was blocked by inhibition of Nrf2 and β-catenin (Figure 7N). BrdU incorporation assays revealed that fisetin significantly increased the proliferation of D580 SYFs compared to control, whereas inhibition of Nrf2 and β-catenin signaling attenuated this effect (Figure 8A,B). Correspondingly, fisetin-induced upregulation of CDK1, CDK2, and Cyclin B1 was markedly reduced by Nrf2 or β-catenin inhibition (Figure 8C–F).

## 4. Discussion

Laying performance is largely governed by the ongoing development of ovarian follicles. In the late-laying period, the transition of prehierarchical follicles into preovulatory follicles in chicken ovaries is disrupted, coupled with an increase in follicular atresia, collectively leading to reduced follicles maturation and ovulation. Previous studies have identified oxidative stress-triggered GCs injury as a major contributor to the arrested development of prehierarchical follicles in aged laying hens [19]. High levels of ROS induce DNA damage and activate the p53/p21/p16 pathways, leading to irreversible cell cycle arrest [20]. Moreover, persistent oxidative damage disrupts mitochondrial function, leading to the loss of ATP production, which is an essential energy supply for cell cycle. In this study, a D-gal-induced senescent GC model was established to investigate how fisetin, a natural flavanol, protects against prehierarchical follicular atresia in aged hens.

D-gal is a simple, convenient, and cost-effective agent widely used to induce senescence in various cells and animal models [21,22]. Our previous study has indicated that D-gal-induced GCs exhibited multiple hallmarks of cellular senescence, such as elevated β-galactosidase activity, reduced cell proliferation, and increased expression of senescence-associated genes including p53, p21, and p16, thereby confirming the suitability of the D-gal-induced model for investigating GCs senescence in laying hens [1]. Fisetin, a plant-derived flavonol, has been well-documented for its diverse pharmacological properties, particularly its antioxidative and anti-aging properties. Previous studies have shown that fisetin can reduce the accumulation of the aging marker SA-β-gal, thereby maintaining human adipose-derived stem cells in a senescent-free state during culture expansion [23]. Furthermore, in aged mice, intermittent supplement with fisetin has been evidenced to improve physical performance and reduce cellular senescence in skeletal muscle [24]. Consistently, our data revealed that fisetin pretreatment markedly alleviated the D-gal-induced decline in cell viability, decreased senescence-associated β-galactosidase activity, and relieved cell cycle arrest, while simultaneously promoting the expression of proliferation-related proteins such as CCND1, CDK2, CDK1, Cyclin B1, and elevating the levels of cell differentiation-related genes in D-gal-induced senescent GCs. Collectively, these findings suggest that fisetin effectively delays GC aging in chickens, providing a mechanistic basis for its potential role in maintaining ovarian function during the late laying period.

Mitochondria, which are abundant organelles in GCs, are responsible for providing sufficient ATP to support cellular growth, proliferation, and division. However, their functional integrity declines progressively with advancing ovarian age. A clinical study suggested that GCs, which isolated from the antral follicles of older women with diminished ovarian reserve, exhibited a reduced proportion of morphologically normal mitochondria, decreased ATP levels, and downregulated expression of mitochondrially encoded genes involved in oxidative phosphorylation [25]. Consistently, in hens, mitochondrial damage such as structural swelling, disorganized cristae, and vacuolation, has been identified in ovarian GCs during the late laying period, ultimately leading to mitochondrial dynamics imbalance and contributing to prehierarchical follicular atresia [26,27]. In our study, D-gal exposure led to mitochondrial impairment in chicken GCs, with a significant reduction in both MMP and ATP levels. Notably, fisetin pretreatment effectively restored MMP, enhanced the expression of mitochondrial-related proteins such as TOMM20, and upregulated key genes associated with mitochondrial biogenesis and energy metabolism, including *TFB2M*, *TFAM*, *POLRMT*, and *ATPase8*. Consequently, ATP levels in GCs were significantly elevated under fisetin intervention upon D-gal stimulation. Oxidative stress acts as a major driver of mitochondrial dysfunction during follicular atresia, arising from excessive ROS accumulation and impaired antioxidant defenses involving enzymes such as T-SOD, CAT, and GSH [28]. Supplementation with antioxidants may improve oxidative stress in the aging ovarian microenvironment, thereby supporting physiological integrity of growing follicles. Recent studies have reported that fisetin supplementation markedly improves oxidative markers, including enhancing CAT, T-SOD enzyme activities, improving GSH concentrations, and inhibiting the accumulation of peroxidation products such as MDA and ROS, thereby alleviating traumatic brain injury, LPS-induced endometritis, and D-gal-triggered oxidative stress and memory deficits in mice [29,30,31]. Consistent with these studies, our data suggested that pretreatment with fisetin significantly suppressed intracellular ROS and MDA accumulation while enhancing activities of antioxidant enzymes CAT and T-SOD in D-gal-induced chicken GCs. Collectively, these results demonstrate that fisetin alleviates oxidative stress, thus maintaining mitochondrial function and ensuring ATP production, which in turn supports the proliferation of D-gal-induced senescent chicken GCs.

As a critical regulator, Nrf2 serves as a master regulator of the cellular antioxidant defense system by regulating the transcription of genes encoding antioxidant enzymes, facilitating the clearance of damaged proteins, and maintaining mitochondrial homeostasis. Accumulating evidence suggests that the antioxidant properties of fisetin across diverse tissues and cell types are largely attributed to its activation of the Nrf2/HO-1 signaling axis. For instance, fisetin demonstrated a therapeutic effect against deep vein thrombosis in mice, whose underlying mechanism involved the activation of Nrf2 signaling and subsequent induction of antioxidant enzyme expression [32]. Furthermore, fisetin suppressed adjuvant-induced oxidative damage associated with rheumatoid arthritis in rats by promoting NQO1 and Nrf2-mediated HO-1 expression [33]. In this study, D-gal treatment significantly reduced the expression of Nrf2/HO-1 pathway-related proteins, including Nrf2, HO-1, and NQO1, in chicken GCs. However, pretreatment with fisetin markedly up-regulated the expression of these proteins even under D-gal-induced senescence. Importantly, inhibition of Nrf2 signaling with ML385 abolished the protective effects of fisetin on the D-gal-induced decline in Nrf2/HO-1 signaling and antioxidant gene expression. These results clearly indicate that fisetin alleviates oxidative stress in D-gal-induced senescent GCs mainly via upregulation of the Nrf2/HO-1 signaling pathway.

As ovarian follicular function declines, the GCs undergo cell cycle arrest, a process closely associated with the suppression of Wnt/β-catenin signaling pathway [34]. β-catenin serves as the central transcriptional effector of Wnt signaling. During G1 phase, the accumulation of β-catenin induces the transition to S phase, upregulates CCND1 expression and further inhibits the expression of the CDK inhibitors p21 and p15 [35]. Conversely, p53 protein serves as a key regulator of cell growth arrest by increasing the expression of the target p21, ultimately leading to G0/G1 cell cycle arrest [36]. Additionally, Lamin B1 loss has been described as a senescence-associated biomarker in vivo and in vitro models for cellular senescence, including D-gal-induced senescence models [37,38]. In this study, we observed that with advancing age in laying hens, the expression of β-catenin, Lamin B1, and CCND1 decreased, whereas the levels of senescence-related genes, including *p53*, *p21*, and *p15*, were significantly upregulated, suggesting that β-catenin levels in GCs is correlated with the follicular atresia. Further investigation revealed that in SYF-GCs, D-gal treatment could reduce the expression of β-catenin; however, the addition of fisetin promoted β-catenin nuclear translocation and upregulated the level of phosphorylated GSK3β and CDK1, while concurrently inhibiting p53 and p21 expression and improving cell cycle arrest, thereby increasing the population of GCs in the G2 phase. In addition, treatment with the β-catenin inhibitor IWR-1 abolished the effects of fisetin to relieve D-gal-induced cell cycle arrest and to downregulate senescence-associated proteins in GCs. These findings indicate that fisetin mitigates senescent SYF-GCs growth arrest mainly via enhancing the expression of nuclear β-catenin and suppressing the CDK inhibitors p53, p21 and p16 transcription. Moreover, co-treated with either the β-catenin inhibitor IWR-1 or the Nrf2 inhibitor ML385 attenuated the protective role of fisetin on the naturally aging chicken SYFs, as evidenced by reduced antioxidant capacity, lower ATP levels, and diminished follicular cell proliferation. Collectively, these results suggest that fisetin prevents chicken prehierarchical follicular atresia by improving oxidative stress and cell cycle arrest via the coordinated activation of nuclear Nrf2 and β-catenin signaling, thereby delaying GC aging.

## 5. Conclusions

**Figure 9:**
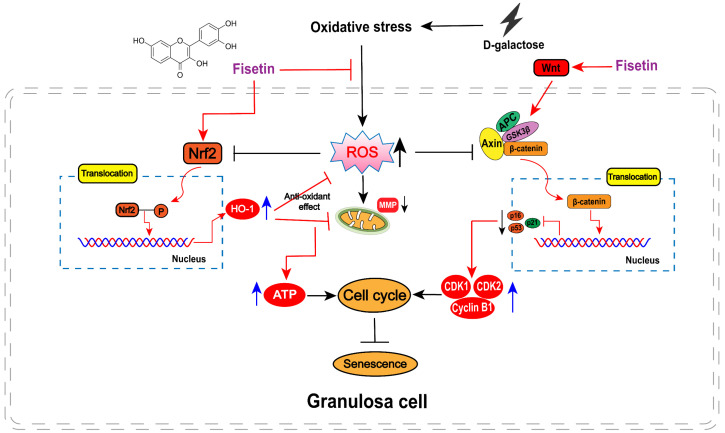
A proposed model of fisetin in alleviating D-gal-induced GCs senescence. Fisetin attenuates oxidative stress and the expression of cell cycle inhibitors in isolated D-gal-induced senescent GCs by activating the Nrf2 and β-catenin nuclear translocation, thereby elevating ATP production and the levels of cell proliferation-related proteins, ultimately improving GCs senescence.

As shown in Figure 9, our data showed that fisetin significantly enhanced the antioxidant capacity of D-gal-induced senescent SYF-GCs by activating the Nrf2/HO-1 signaling pathway, thereby alleviating oxidative damage and promoting intracellular ATP production. Meanwhile, fisetin mitigated cell cycle arrest and reduced the expression of senescence-associated proteins in D-gal-treated GCs through upregulated β-catenin nuclear translocation. These findings provide novel insights into the cellular level mechanisms by which fisetin attenuates prehierarchical follicular atresia in laying hens during the late laying period, highlighting its potential as a nutritional strategy to delay ovarian aging and sustain reproductive performance.

## Data Availability

The data supporting the conclusions of this article will be made available by the authors, without undue reservation.

## Supplementary Materials

The following supporting information can be downloaded at: https://www.mdpi.com/article/10.3390/cells14211704/s1, Table S1: Primers used for qRT-PCR.
