# Supplementary material for: Preventive Effect of Fisetin on Follicular Granulosa Cells Senescence via Attenuating Oxidative Stress and Upregulating the Wnt/β-Catenin Signaling Pathway
Source: Cells. 2025 Oct 30;14(21):1704. doi: 10.3390/cells14211704 (PMC12610666; doi:10.3390/cells14211704)
Supplement: Supplementary file 1 [file cells-14-01704-s001.zip › cells-3922486-supplementary.pdf]

Supplementary Data

Table S1. Sequences of the PCR primers.

Gene name	Accession number	Primer sequence (5'-3')	Product size (bp)
Cyp11a1	NM_001001756.2	F: GGTGGCATACCGTGACTACC<br>R: ACAAAGTCCTGGCTCACCTG	159
StAR	AF220436.1	F: GTGATGGCCCTTATCTCGGT<br>R: TGGTGGCTGCTACAAACACT	131
Axin	AY640375.1	F: GCGGAAGAAGAAGGCGAGGATG<br>R: AGTGATGAAAAGGTTGGGCGGAAG	88
β-catenin	U82964.2	F: AGTCATTGGCAGCAGCAGTCATATC<br>R: TTGCGTTGTGTCCACATCTTCCTC	117
SIRT1	NM_001004767.2	F: GTTGCTTCTCCAAGATGGCG<br>R: CCGTCTTCCGAGTTCAGGC	124
p21	AF513031.1	F: CAAATGTGCTGAAGGTGCCC<br>R: CCAGCCATGGGTCAACAGAA	84
p53	NM_205264.1	F: GCCGTGGCCGTCTATAAGAA<br>R: GGTCTCGTCGTCGTGGTAAC	159
p15	NM_001008479.2	F: GTAGCTGCACCCTTTTTGGG<br>R: TTATTTGGTCCGGGTGTCCC	71
SOD	NM_205064.1	F: GGCAATGTGACTGCAAAGGG<br>R: CCCCTCTACCCAGGTCATCA	133
CAT	NM_001031215.2	F: TCAGGAGATGTGCAGCGTTT<br>R: TCTTACACAGCCTTTGGCGT	109
Mgst	NM_204794.2	F: AAAGAACAGAAACCCCATTCAG<br>R: GCACAGAGGGACATTTTGATT	82
Gsr	XM_015276627.1	F: TCCTGACTACGGCTTCGAGA<br>R: AACTTGCCGTAACCACGGAT	150
Gsta	NM_204818.2	F: GCAGAGCCATCCTCAGCTAC<br>R: CCTTTGCCTCAGGTGGAGAG	150
FSHR	NM_205079.2	F: AGGTCTTCGCACTCCTTCCTTCTAG<br>R: GGAGAATCAGCAAGCAGGTGAGAC	92
LIF	XM_040685142.2	F: GGAGAGCAGCAAGGACAAGAGC<br>R: GTGAGGTAGGAGGACACGAGCAG	109
LIFR	XM_046934683.1	F: TGCCTGGAGCCGAGTAGTAGTTC<br>R: ACATCCGTTGTATCTGCTGTCCTTC	143
COX2	NM_001167719.1	F: CTGCTCCCTCCCATGTCAGA<br>R: CACGTGAAGAATTCCGGTGTT	123
Sp1	NM_204604.2	F: CAAGGAGCAGGGCAGCAATGG<br>R: CAGGTTGGAAGCAGCCGTGAC	94
GATA6	XM_046910483.1	F: TCTGTATGCCTCCTAGCGGTACG<br>R: TTCCACACATTAGCCACTGCCAAG	133
IGFR1	NM_205032.3	F: GTGGTTCGCCTGCTAGGTGTTG<br>R: CTTCTTCAGAGTTGGAGGTGCTTGG	150

INHBA	NM_001396543.1	F: TGTGCGGATTGCCTGTGACTTG<br>R: GCGATTGTTCCTTCTCCTCTTCTCC	143
NR5A1	XM_046928755.1	F: TCCTCCACCATCCACAGCATCC<br>R: CGTTCATAGTCAGCAGGCGTCAG	83
TFB2M	XM_040666918.2	F: AAACCACCTGCTGTGTGTTCTGAC<br>R: GTAGCCTCCAAAGTGTGTTCCTCTC	136
TFAM	XM_046919713.1	F: TCCGCTTCCTGAGGGACAACC<br>R: GCCAGTCTGTCTTCCTTGCTTCC	150
POLRMT	XM_040692726.2	F: TAGGAAGAAGGATGCCGAGGAAGG<br>R: CCGTCATCACCGTCTGCTTCAC	92
ATPase8	AB233988.1	F: CCTGAACCTGACCATGAACCTAAGC<br>R: GAGAGGCGGTTGTTGATCCATCG	144
p16	NM_204434.1	F: GAAGCGCGGAAGAAGACACC<br>R: GGCAACCGACGGAATGTTTG	123
β-actin	*NM_205518.2*	F: CCAGCCATGTATGTAGCCATCCAG<br>R: GGTAACACCATCACCAGAGTCCATC	93

Cyp11a1: cytochrome P450 family 11 subfamily A member 1; *StAR:* steroidogenic acute regulatory protein; *Axin:* axin-related protein transcript variant 2; *SIRT1:* sirtuin 1; *SOD:* superoxide dismutase; *CAT:* catalase; *Gsr:* glutathione reductase; *Gsta:* glutathione S-transferase; *FSHR:* follicle stimulating hormone receptor; *LIF:* leukemia inhibitory factor; *LIFR:* LIF receptor; *COX2:* prostaglandin-endoperoxide synthase 2; *GATA6:* GATA binding protein 6; *IGFR1:* insulin like growth factor 1 receptor; *INHBA:* inhibin beta A subunit; *NR5A1:* nuclear receptor subfamily 5 group A member 1; *TFB2M:* transcription factor B2, mitochondrial; *TFAM:* transcription factor A, mitochondrial; *POLRMT:* RNA polymerase mitochondrial.
